# Fermentation, Handling and Claimed Therapeutic Effects of *Dhanaan*: An Ethiopian Fermented Camel Milk

**DOI:** 10.1002/fsn3.70295

**Published:** 2025-05-19

**Authors:** Tiruha H. Karssa, Jamal B. Kussaga, Teresa Semedo‐Lemsaddek, Jovin K. Mugula

**Affiliations:** ^1^ Department of Food Science and Agro‐Processing Sokoine University of Agriculture Morogoro Tanzania; ^2^ SACIDS Africa Centre of Excellence for Infectious Diseases SACIDS Foundation for One Health, Sokoine University of Agriculture Morogoro Tanzania; ^3^ Department of Biology Hawassa University Hawassa Ethiopia; ^4^ CIISA—Centre for Interdisciplinary Research in Animal Health, Faculty of Veterinary Medicine University of Lisbon Lisbon Portugal; ^5^ Associate Laboratory for Animal and Veterinary Sciences (AL4AnimalS) Lisbon Portugal; ^6^ BioISI—Biosystems and Integrative Sciences Institute, Faculty of Sciences University of Lisbon Lisbon Portugal

**Keywords:** camel milk, *Dhanaan*, Ethiopia, therapeutic effect, traditional fermentation

## Abstract

*Dhanaan* is a spontaneously fermented camel milk that is popular in the Somali regional state of Ethiopia and plays a significant role in the livelihoods of pastoral communities. This study aimed to investigate traditional fermentation methods of *dhanaan* in these communities. Four districts, namely, Gursum, Shabelle, Degehabur, and Birqot, were purposively selected on the basis of camel rearing, camel milk production, and consumption. A cross‐sectional survey of traditional fermentation methods, handling, utilization, and the claimed therapeutic effects of *dhanaan* was conducted using both open‐ and closed‐ended questions in the districts. The traditional preparation of *dhanaan* involves spontaneous fermentation of camel milk at ambient conditions for approximately 48–72 h in smoked plastic jerry cans (90%). In most districts, *dhanaan* preparation was done on a weekly basis (> 50%), except in Birqot, where the majority (54%) of participants prepared it on a daily basis. The majority of participants (> 95%) had never attended any food hygiene training programs. Over 80% of the participants consumed *dhanaan* as a traditional medicine to treat conditions such as diarrhea, abdominal pain, constipation, gastric pain, fever, and diabetes. In conclusion, *dhanaan* preparation requires the use of best practices if the quality and safety of products are to be guaranteed.

## Graphical abstract

*Dhanaan* is a spontaneously fermented camel milk that is popular in the Somali regional state of Ethiopia. The traditional preparation of *dhanaan* involves spontaneous fermentation of camel milk at ambient conditions for approximately 48–72 h in smoked plastic jerry cans. It is also consumed as traditional medicine to treat conditions such as diarrhea, abdominal pain, constipation, gastric pain, fever, and diabetes. *Dhanaan* preparation requires the use of best practices if the quality and safety of the products are to be guaranteed.

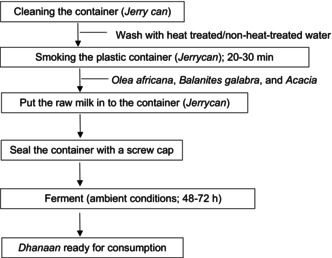

## 1 Introduction

Fermented foods play a vital role in the human diet in different parts of the world. Since the beginning of human civilization, natural fermentation has been practiced as a strategy to either extend the shelf life of perishable products or for culinary purposes. Africa is endowed with a wide range of fermented foods, including cereal‐based (Franz et al. 2014), legume‐based (Obafemi et al. 2022), dairy‐based (Agyei et al. 2020), and fruit‐based (Misihairabgwi and Cheikhyoussef 2017) foods which have major impacts on health, nutrition, and socioeconomic status across the continent. Fermented milk products have been reported to have anticancer effects (García‐Burgos et al. 2020), increase bone mineral density (Ong et al. 2020), improve lactose tolerance (Saleem et al. 2024), lower cholesterol (Yerlikaya 2023), increase nutrient availability (García‐Burgos et al. 2020), and increase the immune system (Santiago‐López et al. 2015). Although African fermented milk products may undergo similar preparation methods, they carry different names in various regions or countries (Agyei et al. 2020). For example, in Ethiopia, fermented dairy products are assigned various names like *ergo* (sour milk), *dhanaan* (fermented camel milk), *ititu* (milk curd), *ayib* (cottage cheese), *neter kibe* (spiced butter), *kibe* (traditional butter), *aguat* (whey) and *arerra* (sour defatted milk) (Berhe et al. 2017).

Camel milk plays an important role in human nutrition, especially in arid and hot regions worldwide (Khaliq et al. 2019; Asres and Yusuf 2014). Camel milk contains all the essential nutrients found in bovine milk, but it also contains many antibacterial substances, vitamin C, and iron (Osman et al. 2024). It can be consumed either in its fresh or fermented form (*dhanaan*). In addition, it has been claimed to have several therapeutic effects, including antidiabetic (Swelum et al. 2021), anticancer (Abrhaley and Leta 2018), antihypertensive (Kocyigit et al. 2024), relief of abdominal pain (Kaskous 2016), bone strength, and diarrhea (Khaliq et al. 2019). Furthermore, camel milk and products (i.e., *dhanaan*) are the main sources of income for many pastoral communities in the Somali regional state.

However, *dhanaan* is prepared mainly when there is a surplus of milk and if pastorals plan to travel long distances, as it has a longer shelf life than fresh milk. *Dhanaan* is prepared by collecting fresh camel milk in a clean and smoked plastic container called a *Jerry can* and then allowed to ferment spontaneously at ambient temperature (25°C–35°C) for approximately 48–72 h without the addition of starter cultures (Berhe et al. 2019). The milk containers used for *dhanaan* fermentation are smoked for 20–30 min and contain glow splinters of *Olea afana* which impart a smoky flavor to the fermenting *dhanaan*.

To date, research has focused on the chemical composition and microbiological quality of *dhanaan* (Biratu and Seifu 2016), its therapeutic value (Asres and Yusuf 2014), handling or preparation, preservation, and utilization (Birhanu et al. 2021; Seifu 2007). However, despite its significance and role in the livelihood of pastoral communities in the Somali regional state of Ethiopia, very little research has been carried out, and there is limited documentation of traditional preparation methods for *dhanaan* and it still needs to be addressed. It is important to investigate the traditional preparation methods for *dhanaan* in order to improve product quality, safety, and commercialization. The study identified gaps in the preparation methods of *dhanaan*, which warrant further action by different stakeholders to ensure food and nutrition security for pastoral communities in the Somali regional state of Ethiopia. Therefore, the aim of this study was to investigate the existing fermentation, handling, and preservation practices of *dhanaan* among the pastoral communities of the Somali regional state in Ethiopia and propose measures for improvement. This survey study is potentially significant, as it involved an on‐site (household survey) investigation of the traditional practices and knowledge of pastoral communities. The survey studies reported so far were collected mainly from open markets, milk producing centers, restaurants, etc.

## 2 Materials and Methods

### 2.1 Study Area

**FIGURE 1:**
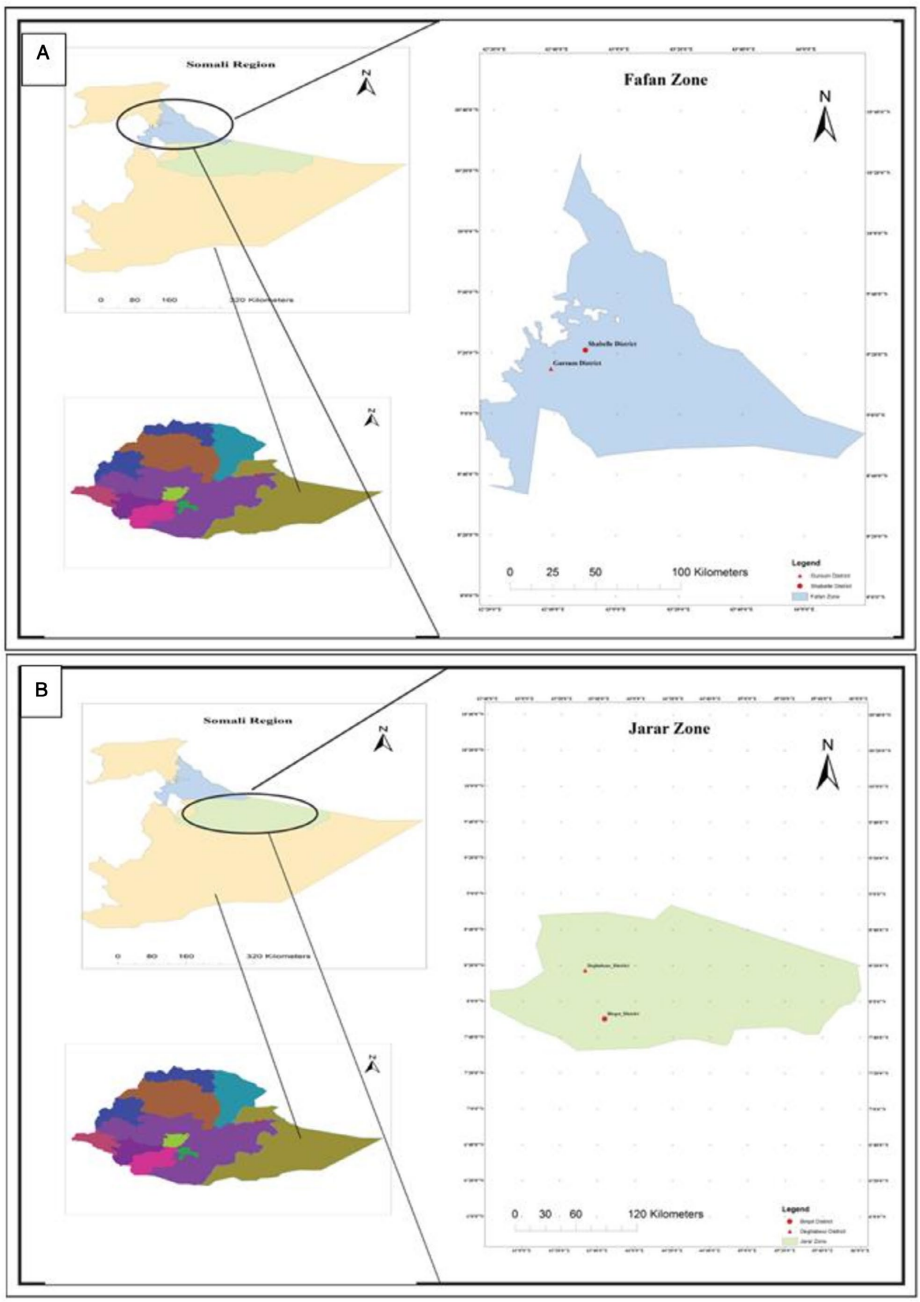
Map of the study area. (A) Shabelle and Gursum districts in the Fafan zone. (B) Degehabour and Birqot districts in the Jarar zone.

This study was conducted in two purposively selected zones (Fafan and Jarar) of the Somali Regional State in Eastern Ethiopia (Figure 1). The two zones are among the nine administrative zones of the Somali region and are characterized by households that keep camels and produce and consume *dhanaan*. Two districts were selected from each zone (Fafan: Gursum and Shabelle; Jarar: Degehabur and Birqot) on the basis of their potential for camel rearing, camel milk production, and consumption.

### 2.2 Identification of Villages and Households

Prior to the main survey, a random visit was arranged with local community leaders, enumerators, and supervisors to identify the study villages in each of the four selected districts. A list of potential villages in each district was prepared and compiled. Ten villages from each district (40 villages in total) were randomly chosen via a lottery system for household selection. In each randomly selected village, households that possessed camels and prepared and consumed *dhanaan* were identified, listed, and compiled for the survey. Then, via the lottery system, potential households were randomly chosen from villages that were previously chosen from the compiled list.

### 2.3 Study Design and Sample Size

A cross‐sectional study design was employed to conduct a household survey between December 2023 and March 2024. The sample size for this survey was determined using Yamane's (1967) formula, which assumes a confidence level of 95% and a proportion of 0.50.

$$n=\frac{N}{1+N{\left(e\right)}^{2}}$$

where *n* = sample size; *N* = population size; 3, 845,822; and *e* = level of precision of the margin of error, 0.05.

By computing the values via the formula, the sample size was 400 in all the selected study villages.

### 2.4 Data Collection Tools

Both open‐ and closed‐ended questionnaires were used to obtain information about the traditional preparation methods, handling methods, utilization, and claimed therapeutic value of camel milk. Interviews and observations were conducted to obtain additional information. The questionnaire was divided into three parts: demographic characteristics; preparation/fermentation of *dhanaan*; and handling, processing, utilization, and claimed medicinal values. To ensure that the questions were simple and reliable, the questionnaire was first prepared in English, then translated into local dialects, and finally back to English. The face and content validity of the questionnaire were assessed by three experts. The data were collected through trained research assistants who spoke the local language. The research assistants in each district read each question to the respondents and wrote the answers in the space provided on the same sheet. Additional discussions and interview responses were recorded in separate notebooks.

### 2.5 Assessment of Traditional Preparation Methods

This section of the questionnaire contained 5 (3 close‐ended, 2 open‐ended) questions on preparation/fermentation of *dhanaan*. The questions included were targeted at ingredients of *dhanaan* preparation, equipment used, frequency of *dhanaan* preparation, and packing of *dhanaan*.

### 2.6 Assessment of Handling Methods, Processing, Utilization, and Claimed Medicinal Values

Questions related to hygienic practices, storage, distribution, selling, utilization, preference, and therapeutic value of *dhanaan* were assessed in this section. It contains 20 questions (17 close‐ended and 3 open‐ended). The main questions included in this section were about previous training in food hygiene, smoking of containers, taking *dhanaan* as a medicine, washing hands before milking, and whether *dhanaan* is their major source of income.

### 2.7 Statistical Analysis

The data were double‐entered into an Excel spreadsheet for cleaning and verification prior to the analysis. Descriptive statistics, including the frequency distribution and percentage of responses, were computed using SPSS (version 29). The associations between some categorical variables and *dhanaan* consumption, preparation, and utilization were determined using Pearson's chi‐square test. Data analysis was conducted at a 95% confidence interval with a margin of error of 5%. Tables, proportions, graphs, and charts were used to present the results. A *p* value of < 0.05 was considered statistically significant.

## 3 Results and Discussion

### 3.1 Demographic Characteristics of the Participants

A total of 400 participants were included in this study. The demographic information of the participants in each district is summarized in Table 1. More than half (58.5%) of the participants were female and between the ages of 15 and 30 years (54.5%). On the other hand, the age group of 65 years and above was the least represented (9%). Gender balance varies from one district to another, as *dhanaan* can be made by both males and females. We randomly asked individuals in the household who had a knowledge of *dhanaan* preparation.

**TABLE 1:** Demographic information of the participants.

	Districts				
	Gursum	Shabelle	Degehabur	Birqot	Total
	% of participants	% of participants	% of participants	% of participants	% of participants
Variables gender					
Male	45	23	57	41	41.5
Female	55	77	43	59	58.5
Age category					
15–30	63	32	44	79	54.5
31–40	21	21	23	8	18.3
41–50	2	16	20	8	11.5
51–60	4	16	5	2	6.8
61 and above	10	15	8	3	9
Education level					
No formal education	91	87	90	43	77.8
1–4 grade	7	13	7	4	7.8
5–8 grade	2	0	2	12	4
9–12 grade	0	0	1	8	2.3
Certificate	0	0	0	2	0.5
Diploma	0	0	0	22	5.5
Degree and above	0	0	0	9	2.3

The majority of the participants (77.75%) had no formal education. This could be due to the fact that the pastoral communities are inhabiting marginal areas where access to school was very minimal. Having some level of education greatly contributes to increasing awareness and understanding, which leads to the application of good hygienic practices during preparation and handling along the supply chain. A study conducted in Kenya by Mogotu et al. (2022) reported that farmers who attended middle and high schools used hygienic practices better than those who had no formal education. In the present study, it has been observed that hygienic practices related to *dhanaan* preparation were poor among pastoral communities in the study area, thereby increasing the risk of foodborne diseases among consumers. This could be due to the fact that most pastoral communities did not attend formal education, the absence of food handling training, scarcity of potable water, and related facilities. A study conducted on metagenomic analysis of bacterial communities of *dhanaan* (Berhe et al. 2019) reported the presence of pathogenic bacteria with major public health concerns.

### 3.2 Preparation of *Dhanaan* at Household Level

All households (100%, Table 2) preferred camel milk to prepare *dhanaan* rather than milk from other animals, as it is associated with many special properties, including medicinal value, long shelf life, high nutritional value, and taste. A similar study conducted in the United Arab Emirates, Cheikh Ismail et al. (2022) reported that consumers prefer camel milk over milk from other animals because of its taste, nutritional value, and therapeutic properties. In addition, camels have the ability to survive in arid and semi‐arid areas, where other livestock species are unable to survive. *Dhanaan* is prepared by placing fresh whole camel milk in a 5‐l screw cap container called a *Jerry can* and allowed to ferment at ambient conditions for approximately 48–72 h. During the survey, it was observed that the use of *jerry cans* by the majority of the respondents was due to a lack of awareness and formal education. The plastic containers used by pastoralists in the study area are difficult to clean and expose milk to further contamination compared to aluminum containers. The majority of the households prepared *dhanaan* weekly in all districts, except Birqot, where 54% of the households prepared *dhanaan* daily (Table 2). Most of the participants in the four districts did not pack *dhanaan*.

**TABLE 2:** Traditional fermentation of *dhanaan*.

	Districts			
	Gursum	Shabelle	Degehabur	Birqot
	% of participants	% of participants	% of participants	% of participants
Variables Ingredients required				
Camel milk	100	100	100	100
Milk of other animals	0	0	0	0
Starter culture	0	0	0	0
Equipment used				
Clay pot	1	0	4	10
Stainless steel vessel	0	0	0	0
Plastic container (*Jerry can*)	99	100	96	90
Frequency of preparation				
Daily	42	0	44	54
Weekly	51	100	56	33
Once in 2 weeks	2	0	0	13
Monthly	5	0	0	0
Pack *dhaanan*				
Yes	1	2	0	0
No	99	98	100	100

### 3.3 Handling Practices of Participants

Food handlers and processing conditions play crucial roles in ensuring food safety during the preparation, storage, and distribution of prepared foods. Almost all (> 95%) of the participants had not attended any food hygiene training (Table 3). Lack of food hygiene training negatively affects the safety of foods, thereby affecting the health of consumers (Birhanu et al. 2021; Tuglo et al. 2021). Most of the participants (> 90%) in the three districts (Shabelle, Degehabur, and Birqot) washed their hands before milking, with the exception of the Gursum district, where 22% of the participants did not wash their hands before milking. However, in a study conducted in the Borena zone of Ethiopia, Teshome et al. (2019) reported that none of the participants practiced hand washing before milking. These findings indicate that milk production, processing, and utilization practices vary according to region, tribe, and culture. In the Shabelle district, almost all participants (99%) used tap water for cleaning hands and milking containers, whereas river water and well water were used by the majority of participants in the Gursum (53%) and Degehabur (31%) districts, respectively, owing to a lack of access to tap water. Furthermore, the majority of the participants in all districts did not apply any water treatment options before use. This might be due to a lack of awareness and training regarding good handling practices of milk. Similarly, in a study conducted in the Gurage Zone of southern Ethiopia, Babege et al. (2020) reported that the water used for cleaning was not treated (not potable).

**TABLE 3:** Handling practices of participants.

Variables	Districts			
	Gursum	Shabelle	Degehabur	Birqot
	% of participants	% of participants	% of participants	% of participants
Previous training on food hygiene				
Yes	3	0	0	0
No	97	100	100	100
Wash your hand before milking				
Yes	78	99	90	93
No	22	1	10	7
Source of water for washing milking equipment				
Tap	29	99	18	53
River	53	0	51	18
Pond	4	0	0	21
Well	14	1	31	8
Boil water before use				
Yes	49	27	3	18
No	51	73	97	82
Smoke containers before milking				
Yes	80	99	82	90
No	20	1	18	10

Smoking of equipment used for milk handling is a common practice in different regions of Ethiopia. Similarly, the majority of the participants in all districts smoked milk containers before use. Smoking improves the flavor and taste of products. It can also improve the shelf life of foods by reducing the number of contaminants or spoilage microorganisms through the antimicrobial effect of plant/wood smoke (Asefa and Abrha 2022; Lemma et al. 2024). Teshome et al. (2019) reported that smoking containers prior to milk processing activities enhances taste and flavor and reduces contamination. However, Babege et al. (2020) reported that 57.8% of the participants did not smoke milk containers.

### 3.4 Utilization and Claimed Therapeutic Value of *Dhanaan*

This survey indicated that > 85% of the participants in all districts preferred the consumption of fresh camel milk over *dhanaan* milk. In Dgehabur (98%) and Birqot (58%) of the participants sell *dhanaan*, whereas 98% in Gursum and 55% in Shabelle did not sell *dhanaan*. This could be explained by less availability of camel milk, and thus pastoralists did not intend to make *dhanaan*. This observation corresponds to a study conducted in the Borana area of southern Ethiopia (Birhanu et al. 2021), which reported that 100% of the participants used fresh camel milk. In general, *dhanaan* is prepared when there is surplus milk to prevent spoilage. Therefore, the consumption of *dhanaan* is limited to dryer seasons, when there is little supply of fresh camel milk. In this survey, it was observed that pastoralists preferred using *dhanaan* because of its longer shelf life (3–5 months) for use in their nomadic movements. Such extended shelf life of *dhanaan* is also crucial for pastoralists living in arid areas where access to refrigeration facilities is not available. It is also an important component of the diet and is consumed by all family members.

More than 80% of the participants consumed *dhanaan* because of its therapeutic value against diarrhea, abdominal pain, stomach problems, constipation, gastric pain, fever, and diabetes. This could be because camels graze on a variety of plant species and active agents that have therapeutic properties and are secreted into the milk (Gebremichael et al. 2019). Various studies have shown that *dhanaan* consumption has therapeutic effects against asthma, jaundice, diarrhea, malaria, and constipation (Birhanu et al. 2021; Asres and Yusuf 2014; Seifu 2007). However, the majority of reports regarding the therapeutic effects of *dhanaan* were survey data obtained from pastoralists, which calls for laboratory‐based experimental research. *Dhanaan* is normally prepared at the household level, often by people without formal education. They were neither inspected nor registered. This situation can jeopardize the quality and safety, commercialization, and medicinal significance of dhanaan. Although *dhanaan* could offer some therapeutic value, if not handled hygienically, it may be contaminated with various health hazards that cause foodborne diseases. A study conducted in Ghana, Tuglo et al. (2021) revealed that those who attended secondary education were aware of good hygienic practices when preparing food. The majority of participants reported that there were no customer complaints except for a few who reported quality (8% in Gursum), (16% in Degehabur), and price (22% in Birqot) (Table 4). Pastoralists strongly believed that there is no foodborne illness as a result of *dhanaan* consumption, except those who reported (Degehabur, 28% and Birqot, 24%) a feeling of suffocation and discomfort for 10–15 min. According to discussions with the participants, the main reason for customer complaints regarding quality is that some households adulterate milk with water, which undermines the quality and safety of *dhanaan* resulting from the use of contaminated water. This could lead to a risk of foodborne diseases for the consumer. Similarly, complaints regarding prices are due to seasonal price variations. A study conducted in Gursum districts, Omer and Ateye (2023) reported that milk prices were higher during the dry season than during the wet season.

**TABLE 4:** Utilization and claimed therapeutic value of *dhanaan*.

Variables	Districts			
	Gursum	Shabelle	Degehabur	Birqot
	% of participants	% of participants	% of participants	% of participants
Form of camel milk consumption				
Fresh	96	96	90	85
*Dhanaan* (fermented)	0	0	8	4
Both fresh and *dhanaan*	4	0	2	11
Personal interest	0	4	0	0
*Dhanaan* for sale				
Yes	2	45	98	58
No	98	55	2	42
Consumers of *dhanaan*				
Infants	0	0	0	0
School going children	0	0	0	0
Adults	1	38	0	48
Every member of the household	99	62	100	52
Take *dhanaan* as a medicine				
Yes	83	100	99	87
No	17	0	1	13
Therapeutic benefits of *dhanaan*				
Diarrhea	49	19	6	26
Abdominal pain	9	11	9	0
Stomach discomfort	24	35	27	21
Constipation	5	12	41	17
Gastric pain	13	8	0	11
Fever	0	13	4	3
Diabetes	0	2	13	22
Conduct quality tests				
Yes	0	0	0	0
No	100	100	100	100
Inspection by the food control authority				
Yes	0	0	0	0
No	100	100	100	100
Registered to produce *dhanaan*				
Yes	0	0	0	0
No	100	100	100	100
Sickness after consuming *dhanaan*				
Yes	0	2	28	24
No	100	98	72	76
Customer complaints				
No complaint	92	100	81	68
Discomfort	0	0	0	0
Price	0	0	3	22
Adulteration	8	0	16	10

### 3.5 Associations Between Demographic Information and *Dhanaan* Preparation, Consumption, and Utilization

This study revealed a significant association (*p* = 0.001) between age and *dhanaan* consumption, with every household member who consumed *dhanaan* being 15–30 years old. This is because individuals in the 15–30 age category are old enough for a nomadic life. The pastoral communities in the study area mainly lead a nomadic life, and they prefer to consume *dhanaan* to sustain their lives in dry areas. In this study, it was difficult to associate the level of education with hygienic practices, containers used, and related practices. This is mainly because the majority of participants in the current study did not have any formal education. However, earlier studies conducted in Ethiopia, Fekadu et al. (2024), Kenya, Mogotu et al. (2022), and Ghana, Tuglo et al. (2021) reported a significant association between the level of education and hygienic practices.

**FIGURE 2:**
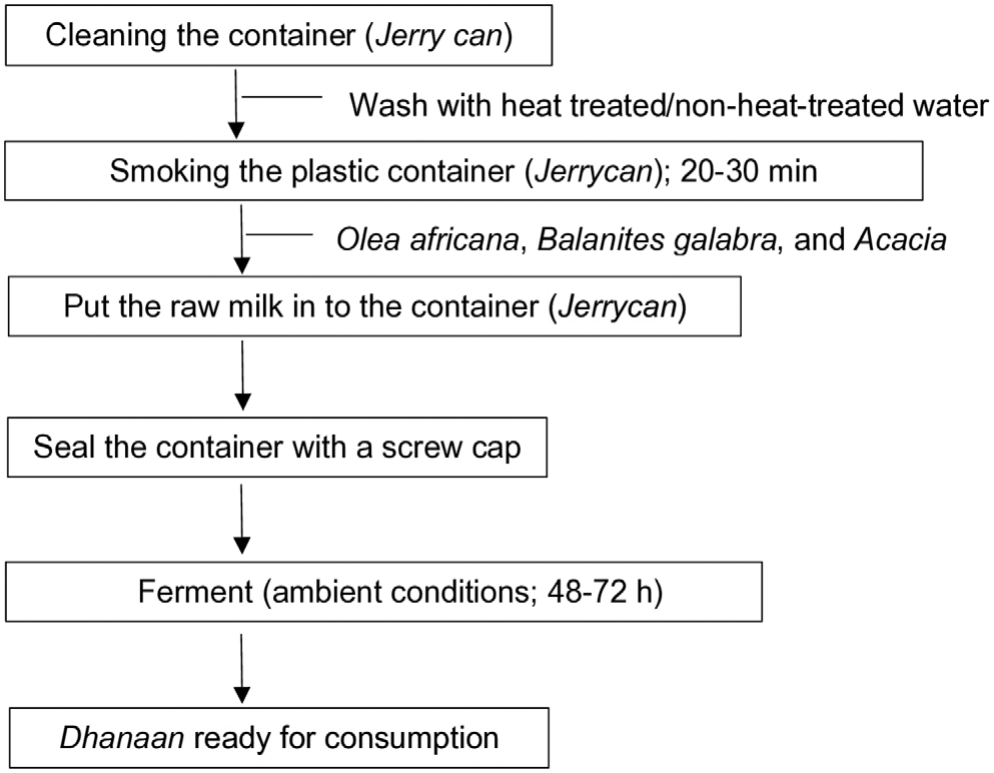
Flow chart of traditional *dhanaan* preparation.

*Dhanaan* is a major fermented camel milk that plays an important role in the livelihood of pastoral communities in rural and urban settlements of the Somali Regional State. The traditional preparation of *dhanaan* involves a few steps for the spontaneous (natural) fermentation of smoked camel milk. It is prepared by putting raw camel milk in a clean and smoked screw‐caped plastic container called a *Jerry can*, followed by screwing the container with the cap, and keeping it at ambient conditions (25°C–35°C) for about 48–72 h (Figure 2; (Berhe et al. 2019)). The pastoral communities pass the smoke in the plastic containers in order to improve the flavor of *dhanaan*. In addition, smoking by using plants, such as *Olea africana* , *Balanites galabra*, and *Acacia*, might extend the shelf life of *dhanaan* due to the probable presence of antimicrobial chemical compounds in the emitted smoke. The antimicrobial effect due to smoking inner surfaces of milk handling containers, as a preservation method in pastoral systems in Kenya, was reported by Wanjala et al. (2016).

## 4 Conclusions

*Dhanaan*, a traditional fermented camel milk product that is a diet and source of income for pastoral communities, is consumed by all age groups in the Somali regional state, Ethiopia. The fermentation process is spontaneously carried out at ambient conditions, approximately 48–72 h. *Dhanaan* is important because of its high nutritional and sensory quality and claimed therapeutic value. The traditional preparation of *dhanaan* does not use best practices, resulting in a product of low quality and safety. Thus, in order to improve the quality and safety, extend shelf life, and commercialize the product, knowledge of the traditional process procedures and identification of critical points is crucial towards the preparation of safer *dhanaan*. Therefore, the processing of *dhanaan* in the study area requires interventions, including raising awareness of good processing practices among the pastoral communities, continuous training, and hygienic control measures that will improve the quality and safety of *dhanaan*. Additionally, there is a need to isolate, identify, and characterize the fermenting microorganisms, including their technological potential to use them as a starter culture for *dhanaan* fermentation. This will enable us to carry out a controlled fermentation, thereby improving the quality and safety of *dhanaan*. This is our next objective in the same research project.

## Author Contributions

**Tiruha H. Karssa:** conceptualization (equal), data curation (equal), formal analysis (lead), funding acquisition (lead), investigation (lead), methodology (equal), project administration (supporting), resources (equal), software (lead), validation (equal), visualization (equal), writing – original draft (lead), writing – review and editing (equal). **Jamal B. Kussaga:** conceptualization (equal), data curation (equal), formal analysis (supporting), funding acquisition (supporting), investigation (supporting), methodology (equal), project administration (lead), resources (equal), software (supporting), supervision (lead), validation (equal), visualization (equal), writing – original draft (supporting), writing – review and editing (equal). **Jovin K. Mugula:** conceptualization (equal), data curation (equal), formal analysis (supporting), funding acquisition (supporting), investigation (supporting), methodology (equal), project administration (lead), resources (equal), software (supporting), supervision (lead), validation (equal), visualization (equal), writing – original draft (supporting), writing – review and editing (equal). **Teresa Semedo‐Lemsaddek:** conceptualization (equal), data curation (equal), formal analysis (supporting), funding acquisition (supporting), investigation (supporting), methodology (equal), project administration (lead), resources (equal), software (supporting), supervision (lead), validation (equal), visualization (equal), writing – original draft (supporting), writing – review and editing (equal).

## Ethics Statement

The authors have nothing to report.

## Consent

All authors read and approved the manuscript for publication.

## Conflicts of Interest

The authors declare no conflicts of interest.

## Data Availability

The data are available upon reasonable request from the corresponding author.

## References

[fsn370295-bib-0001] Abrhaley, A. , and S. Leta . 2018. “Medicinal Value of Camel Milk and Meat.” Journal of Applied Animal Research 46, no. 1: 552–558. 10.1080/09712119.2017.1357562.

[fsn370295-bib-0002] Agyei, D. , J. Owusu‐Kwarteng , F. Akabanda , and S. Akomea‐Frempong . 2020. “Indigenous African Fermented Dairy Products: Processing Technology, Microbiology and Health Benefits.” Critical Reviews in Food Science and Nutrition 60, no. 6: 991–1006. 10.1080/10408398.2018.1555133. 30668144

[fsn370295-bib-0003] Asefa, Z. , and E. Abrha . 2022. “Effect of Traditional Smoking and Cleaning of Milk Equipment With Different Plants and Herbs on Keeping Quality and Organoleptic Properties of Raw Milk in Ethiopia.” International Journal of Applied Sciences & Engineering 9, no. 1: 26–30.

[fsn370295-bib-0004] Asres, A. , and M. Yusuf . 2014. “Traditional Consumption, Therapeutic Value and Its Derived Dairy Products of Dromedary Camel (Camelus Dromedaries) Milk in Somali Regional State, Eastern Ethiopia; A Review.” 4, no. 25: 140–146.

[fsn370295-bib-0005] Babege, K. , M. Eshetu , and F. Kassa . 2020. “Hygienic Production Practices and Microbial Quality of Cow Milk in Cheha District of Gurage Zone, Southern Ethiopia.” Open Journal of Animal Sciences 10, no. 3: 592–607. 10.4236/ojas.2020.103038.

[fsn370295-bib-0006] Berhe . 2017. “Traditional Fermented Dairy Products of Ethiopia: A Review.” East African Journal of Sciences 11, no. 2: 73–80.

[fsn370295-bib-0007] Berhe, T. , R. Ipsen , E. Seifu , M. Y. Kurtu , A. Fugl , and E. B. Hansen . 2019. “Metagenomic Analysis of Bacterial Community Composition in Dhanaan: Ethiopian Traditional Fermented Camel Milk.” FEMS Microbiology Letters 366, no. 11: fnz128. 10.1093/femsle/fnz128. 31183493

[fsn370295-bib-0008] Biratu, K. , and E. Seifu . 2016. “Chemical Composition and Microbiological Quality of Dhanaan: Traditional Fermented Camel Milk Produced in Eastern Ethiopia.” International Food Research Journal 23, no. 5: 2223–2228.

[fsn370295-bib-0009] Birhanu, B. , O. Anaf , K. Adem , and H. Beshir . 2021. “Indigenous Knowledge on Camel Milk and Camel Milk Products Hygienic Handling, Processing and Utilization in Borana Area, Southern Ethiopia.” Journal of Food Science and Nutrition Therapy 7, no. 1: 025–032. 10.17352/jfsnt.000029.

[fsn370295-bib-0010] Cheikh Ismail, L. , T. M. Osaili , M. N. Mohamad , et al. 2022. “Camel Milk Consumption Patterns and Perceptions in the UAE: A Cross‐Sectional Study.” Journal of Nutritional Science 11: 1–9. 10.1017/jns.2022.55. PMC9305078 35912304

[fsn370295-bib-0011] Fekadu, Y. , M. Z. Kinde , G. G. Dagnaw , B. Dessalegn , H. Dejene , and A. T. Gessese . 2024. “Knowledge, Attitude, and Practices on Food Safety Among Food Handlers Working in Public Food Service Establishments in Lemi Kura Subcity, Addis Ababa, Ethiopia.” BioMed Research International: 1–14. 10.1155/2024/2675894. PMC10827374 38292064

[fsn370295-bib-0012] Franz, C. M. A. P. , M. Huch , J. M. Mathara , et al. 2014. “African Fermented Foods and Probiotics.” International Journal of Food Microbiology 190: 84–96. 10.1016/j.ijfoodmicro.2014.08.033. 25203619

[fsn370295-bib-0013] García‐Burgos, M. , J. Moreno‐Fernández , M. J. M. Alférez , J. Díaz‐Castro , and I. López‐Aliaga . 2020. “New Perspectives in Fermented Dairy Products and Their Health Relevance.” Journal of Functional Foods 72: 104059. 10.1016/j.jff.2020.104059.

[fsn370295-bib-0014] Gebremichael, B. , S. Girmay , and M. Gebru . 2019. “Camel Milk Production and Marketing: Pastoral Areas of Afar, Ethiopia.” Pastoralism 9, no. 1: 10. 10.1186/s13570-019-0147-7.

[fsn370295-bib-0015] Kaskous, S. 2016. “Importance of Camel Milk for Human Health.” Emirates Journal of Food and Agriculture 28, no. 3: 158–163. 10.9755/ejfa.2015-05-296.

[fsn370295-bib-0016] Khaliq, A. , M. Farhan , J. Chughtai , et al. 2019. “Camel Milk: Massive Paragon of Nutritional and Therapeutic Potentials: A Review.” International Journal of Research Studies in Biosciences 7, no. 9: 12–26. 10.20431/2349-0365.0709002.

[fsn370295-bib-0017] Kocyigit, E. , R. Abdurakhmanov , and B. F. Kocyigit . 2024. “Potential Role of Camel, Mare Milk, and Their Products in Inflammatory Rheumatic Diseases.” Rheumatology International 44, no. 3: 425–434. 10.1007/s00296-023-05516-x. 38183445 PMC10867071

[fsn370295-bib-0018] Lemma, H. , L. Asefa , T. Gemechu , et al. 2024. “Indigenous Knowledge on the Practice of Milk Container Fumigation and Its Effect on Microbial Safety of Milk Among Pastoral Communities in West Guji Zone, Southern Ethiopia.” Heliyon 10, no. 4: e25877. 10.1016/j.heliyon.2024.e25877. 38384507 PMC10878909

[fsn370295-bib-0019] Misihairabgwi, J. , and A. Cheikhyoussef . 2017. “Traditional Fermented Foods and Beverages of Namibia.” Journal of Ethnic Foods 4, no. 3: 145–153. 10.1016/j.jef.2017.08.001.

[fsn370295-bib-0020] Mogotu, M. W. , G. O. Abong , J. Mburu , and O. A. Ndambi . 2022. “Assessment of Hygiene Practices and Microbial Safety of Milk Supplied by Smallholder Farmers to Processors in Selected Counties in Kenya.” Tropical Animal Health and Production 54: 4. 10.1007/s11250-022-03214-7. PMC9239957 35764898

[fsn370295-bib-0021] Obafemi, Y. D. , S. U. Oranusi , K. O. Ajanaku , P. A. Akinduti , J. Leech , and P. D. Cotter . 2022. “African Fermented Foods: Overview, Emerging Benefits, and Novel Approaches to Microbiome Profiling.” NPJ Science of Food 6, no. 1: 15. 10.1038/s41538-022-00130-w. 35181677 PMC8857253

[fsn370295-bib-0022] Omer, A. G. , and M. D. Ateye . 2023. “Assessment of Market and Marketing System of Raw Camel Milk in Gursum District, Somali Regional State, Ethiopia.” Asian Journal of Advances in Research 6, no. 1: 484–491.

[fsn370295-bib-0023] Ong, A. M. , K. Kang , H. A. Weiler , and S. N. Morin . 2020. “Fermented Milk Products and Bone Health in Postmenopausal Women: A Systematic Review of Randomized Controlled Trials, Prospective Cohorts, and Case‐Control Studies.” Advances in Nutrition 11, no. 2: 251–265. 10.1093/advances/nmz108. 31603185 PMC7442363

[fsn370295-bib-0024] Osman, H. I. I. , E. T. S. Shuiep , and I. E. M. El Zubeir . 2024. “Chemical Composition of Gariss Produced From Milk of Camels With Different Watering Intervals Using Some Traditional Containers in Al‐Koma Locality, North Darfur State, Sudan.” Journal of Ethnic Foods 11, no. 1: 1–10. 10.1186/s42779-023-00217-z.

[fsn370295-bib-0025] Saleem, G. N. , R. Gu , H. Qu , et al. 2024. “Therapeutic Potential of Popular Fermented Dairy Products and Its Benefits on Human Health.” Frontiers in Nutrition 11: 1–17. 10.3389/fnut.2024.1328620. PMC10933135 38481973

[fsn370295-bib-0026] Santiago‐López, L. , A. Hernández‐Mendoza , H. S. Garcia , V. Mata‐Haro , B. Vallejo‐Cordoba , and A. F. González‐Córdova . 2015. “The Effects of Consuming Probiotic‐Fermented Milk on the Immune System: A Review of Scientific Evidence.” International Journal of Dairy Technology 68, no. 2: 153–165. 10.1111/1471-0307.12202.

[fsn370295-bib-0027] Seifu, E. 2007. “Handling, Preservation and Utilization of Camel Milk and Camel Milk Products in Shinile and Jijiga Zones, Eastern Ethiopia.” Livestock Research for Rural Development 19, no. 6: 15.

[fsn370295-bib-0028] Swelum, A. A. , M. T. El‐Saadony , M. Abdo , et al. 2021. “Nutritional, Antimicrobial and Medicinal Properties of Camel's Milk: A Review.” Saudi Journal of Biological Sciences 28, no. 5: 3126–3136. 10.1016/j.sjbs.2021.02.057. 34025186 PMC8117040

[fsn370295-bib-0029] Teshome, B. , M. Wassie , E. Ayinalem , and G. Tefera . 2019. “Traditional Knowledge of Milk Production, Processing and Utilization in Borena Zone, Ethiopia.” World Journal of Dairy & Food Sciences 14, no. 2: 210–221. 10.5829/idosi.wjdfs.2019.210.221.

[fsn370295-bib-0030] Tuglo, L. S. , P. D. Agordoh , D. Tekpor , Z. Pan , G. Agbanyo , and M. Chu . 2021. “Food Safety Knowledge, Attitude, and Hygiene Practices of Street‐Cooked Food Handlers in North Dayi District, Ghana.” Environmental Health and Preventive Medicine 26, no. 1: 1–13. 10.1186/s12199-021-00975-9. 33941082 PMC8091506

[fsn370295-bib-0031] Wanjala, N. W. , J. W. Matofari , and J. M. Nduko . 2016. “Antimicrobial Effect of Smoking Milk Handling Containers' Inner Surfaces as a Preservation Method in Pastoral Systems in Kenya.” Pastoralism 6, no. 1: 1–7. 10.1186/s13570-016-0064-y.

[fsn370295-bib-0032] Yerlikaya, O. 2023. “A Review of Fermented Milks: Potential Beneficial Effects on Human Nutrition and Health.” African Health Sciences 23, no. 4: 498–507. 10.4314/ahs.v23i4.54. 38974284 PMC11225442

